# Tumor suppressing effects of tristetraprolin and its small double‐stranded RNAs in bladder cancer

**DOI:** 10.1002/cam4.3622

**Published:** 2020-12-01

**Authors:** Wen Jiang, Dandan Zhu, Chenghe Wang, Yu Zhu

**Affiliations:** ^1^ Department of Urology Ruijin Hospital Shanghai Jiao Tong University School of Medicine Shanghai China

**Keywords:** 3′ untranslated region, bladder cancer, cyclin‐dependent kinase 1, small double‐stranded RNAs, tristetraprolin

## Abstract

Bladder cancer (BCa) is a common malignant tumor of urinary system with few treatments, so more useful therapeutic targets are still needed. Antitumor effects of tristetraprolin (TTP) have been explored in many type tumors, but its roles in bladder cancer are still unknown until now. In this study, public expression profiles and tissue microarray analysis showed that TTP mRNA and protein levels decreased in BCa relative to the normal bladder tissue. To explore biological functions of TTP in BCa, 488 TTP target genes, which could be both suppressed and bound by TTP, were identified by comprehensively analyzing publicly available high‐throughput data obtained from Gene Expression Omnibus (GEO). Gene enrichment analysis showed that these genes were enriched in pathways such as cell cycle, epithelial to mesenchymal transition (EMT), and Wnt signaling. Clustering analysis and gene set variation analysis indicated that patients with high expression of TTP target genes had poorer prognosis and stronger tumor proliferation ability relative to the BCa patients with low expression of TTP target genes. In vitro experiments validated that TTP could suppress proliferation, migration, and invasiveness of BCa cells. And TTP could suppress mRNA expression of cyclin‐dependent kinase 1 (CDK1) in BCa cells by target its 3′ UTR. Then, we identified a new small double‐stranded RNA (dsRNA) named dsTTP‐973 which could increase TTP expression in BCa cells, in vivo and in vitro experiments revealed that dsTTP‐973 could suppress aggressiveness of BCa. In conclusion, TTP played a role of tumor suppressor gene in BCa like other tumors, and its dsRNA named dsTTP‐973 could induce TTP expression in BCa and suppress aggressiveness of BCa. With the help of materials science, dsTTP‐973 may become a potential treatment for BCa in the future.

## Graphical abstract

TTP overexpression can inhibit the malignant biological behaviors of BCa, and can be a potential therapeutic target for BCa. dsTTP‐973 which can induce TTP expression in BCa is able to suppress BCa progression, and dsTTP‐973 may be a potential treatment for BCa in the future.

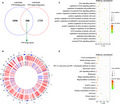

## 1 BACKGROUND

Bladder cancer (BCa) is one of the malignancies that originate from the urinary system, with approximately 550,000 new cases diagnosed and 200,000 deaths occurred globally in 2018.^1^ Compared with other cancer patients, the overall survival (OS) of BCa patients has not significantly improved over the past 30 years due to the lack of new treatment options.^2^ According to the depth of tumor invasion, BCa can be divided into two classifications: non‐muscle invasive BCa (NMIBC) and muscle‐invasive BCa (MIBC).^3^ Patients with NMIBC have high probabilities of recurrence and progression several years after systematic antitumor treatments including transurethral resection (TURB), intravesical chemotherapy, and bacillus Calmette–Guérin (BCG) immunotherapy.^2^, ^4^ And patients with MIBC have high risk of metastasis which cause more than 50% of tumor‐related deaths.^5^ Therefore, more new therapeutic targets and strategies which can be used for curing BCa are still urgent to be found.

Tristetraprolin (TTP) is an important RNA‐binding protein which can destabilize mRNAs of target genes through interacting with AU‐rich elements (AREs) in their 3′ untranslated regions (3′ UTR).^6^, ^7^ TTP has been reported to be downregulated in several tumors such as glioma,^8^ colon cancer,^9^ gastric cancer,^10^ and liver cancer,^11^ and emerging evidences show that TTP can act as a tumor suppressor via destabilizing mRNAs of downstream target oncogenes. For example, TTP was able to inhibit tumor cell growth through mediating mRNA decay of several proliferation‐related genes such as cyclin B1,^12^ cyclin D1, c‐Myc,^13^ and E2F1.^14^ TTP could inhibit angiogenesis by negatively regulating mRNA of vascular endothelial growth factor (VEGF) in colon cancer.^9^ Loss of TTP could increase mRNA stabilization of MMP9, MMP2, and IL‐6 and promote invasiveness of head and neck cancer.^15^ Therefore, induction of TTP expression in tumors may be a promising method for curing cancers.

Small double‐stranded RNAs (dsRNAs) which consist of 21 nucleotides are newfound strategies for specific gene reactivation at the transcriptional level in the past few years.^16^ By forming a complex with Argonaute 2 (Ago2) protein, dsRNAs can specifically bind to DNA of target promoters based on the base complementary pairing principle, and promote formation of RNA‐induced transcriptional activation (RITA) complex to induce transcription of target genes.^17^ Recently, dsRNAs had been shown to reactivate several tumor suppressor genes such as P53,^18^ P21,^16^, ^17^ and VHL,^19^ and significantly inhibited growth and metastasis of cancer cells. With the development of material science, a liposomal nanoparticles loaded dsRNAs named MTL‐CEBPA had been successfully delivered to rat liver and suppressed tumorigenesis of rat hepatocellular carcinoma model via activating CEBPA expression, and the MTL‐CEBPA is now in a phase I clinical trial for patients with advanced cirrhotic hepatocellular carcinoma.^20^ But so far investigations and applications of dsRNAs are still few relative to those of small interfering RNA (siRNA), it is necessary to identify more potential dsRNAs for antitumor therapy.

Anticancer activities of TTP have been validated in many other tumors, but biological functions of TTP in BCa have not been investigated until now. In this study, using bioinformatic analysis and in vitro experiments, we found that TTP also acted as a tumor suppressor in BCa and could be a potential therapeutic target for BCa. Then, we designed a dsRNA called dsTTP‐973 which could induce TTP expression in BCa cells, and found that dsTTP‐973 could significantly suppress aggressiveness of BCa in vitro and in vivo. With the help of materials science, dsTTP‐973 may become a potential treatment for BCa in the future.

## 2 METHODS

### 2.1 Data obtaining

RNA‐seq data of 27 tumors and their corresponding normal tissues were downloaded from The Cancer Genome Atlas (TCGA, https://portal.gdc.cancer.gov/) and The Genotype‐Tissue Expression (GTEx, https://www.gtexportal.org). RNA‐seq data of 25 bladder cell lines were downloaded from Cancer Cell Line Encyclopedia (CCLE, https://portals.broadinstitute.org/ccle). Microarray data of BCa (GSE13507) were obtained from Gene Expression Omnibus (GEO, https://www.ncbi.nlm.nih.gov/gds/).^21^ TTP overexpression microarray data sets (GSE53183) and Photoactivatable Ribonucleoside‐Enhanced Crosslinking and Immunoprecipitation (PAR‐CLIP) sequencing data (GSE53184) were downloaded from GEO. The microarray data sets included transcriptome data of five groups of TTP overexpression cells and control cells, and the PAR‐CLIP sequencing data provided the cDNA sequences which could be in vivo bound by FLAG/HA‐tagged TTP.^22^

### 2.2 Functional and pathway enrichment analysis, cluster analysis, and gene set variation analysis (GSVA)

As an RNA‐binding protein, TTP can regulate cell biological functions via directly inducing mRNA decay of downstream target genes, so we can predict biological functions of TTP via analyzing its target genes.^6^, ^22^, ^23^, ^24^ Then, TTP target genes were identified by comprehensively analyzing microarray data (GSE53183) and PAR‐CLIP sequencing data (GSE53184). For microarray data, fold change (FC) and P value of each gene was generated by comparing mRNA expression levels of genes in control group and those of genes in TTP overexpression group using limma R package, and genes with Log2FC < −1 and *p* value <0.05 were defined as TTP suppressing genes.^25^ For PAR‐CLIP sequencing data, genes with at least one binding site in their 3′ UTRs genes were defined as TTP binding genes. Finally, TTP target genes were defined as the genes that belonged to both TTP suppressing genes and TTP binding genes. Then, enrichment analysis was performed to explore whether these genes were enriched in previous defined Gene Ontology (GO) terms and Kyoto Encyclopedia of Genes and Genomes (KEGG) pathways using clusterProfiler R package, and significant GO terms and KEGG pathways were identified with adjusted *p* < 0.05.^26^ TCGA‐BLCA patients were divided into different groups using clustering analysis according to the expression levels of TTP target genes. About 50 hallmark gene sets of common biological pathways were downloaded from Molecular Signatures Database (http://software.broadinstitute.org/gsea/msigdb), and enrichment scores of the 50 pathways for each TCGA sample were generated using the GSVA R package.^27^ To identify different biological pathways between the two groups, enrichment scores of the 50 pathways in the two groups were compared using Mann‐Whitney *U* test.

### 2.3 Tissue microarray

The paraffin‐embedded tissue microarray containing primary BCa samples (n = 59) and normal bladder tissue (n = 18) was purchased from Shanghai outdo biotech company. We confirmed that the specimens were collected in accordance with recognized ethical guidelines by checking informed consent and ethical approval document provided by the company. Meanwhile, this experimental scheme was also approved by the Ethics Committee of Ruijin Hospital.

### 2.4 Immunohistochemical assay (IHC)

Target proteins of paraffin sections were stained using IHC methods as described in previous study.^28^ Expression levels of target proteins were evaluated by two independent pathologists according to the scoring criteria as follow: the proportion of positive cells was 0%–1% = 0 points, 1%–25% = 1 point, 26%–50% = 2 points, 51%–75% = 3 points, and 76%–100% = 4 points; then, staining densities were no staining = 0 points, low staining = 1 points, moderate staining = 2 points, and high staining = 3 points; and the total scores=staining area score × staining density score.^29^

### 2.5 Cell culture

The human BCa cell lines 5637 and UMUC3 were purchased from the Cell Bank of the Chinese Academy of Sciences (Shanghai, China) and cultured at 37°C with an atmosphere of 5% CO_2_. The 5637 cells were cultured in 1640 (Gibco, USA) supplemented with 10% fetal bovine serum (FBS, Gibco, USA) and 1% penicillin‐streptomycin, the UMUC3 cells were cultured in minimum Eagle's medium (MEM) (Gibco, USA) supplemented with 10% FBS and 1% penicillin‐streptomycin.

### 2.6 RNA extraction, reverse transcription, and quantitative real‐time PCR (qRT‐PCR)

Total RNA of BCa cells was extracted using TRIzol reagent (Invitrogen, USA) according to the manufacturer's protocol. Takara reverse transcription kit with gDNA eraser (Takara, Japan) was used to remove genomic DNA and reversely transcribe the total RNA into cDNA. cDNAs of target genes were detected using SYBR Premix Ex Taq (Tli RNaseH Plus) (Takara, Japan) kit on the ABI 7500 instrument. All the primer sequences were listed in Table S1. The relative mRNA expressions of target genes were calculated using the 2^−ΔΔCt^ method, with GAPDH (glyceraldehyde‐3‐phosphate dehydrogenase) used as internal control.

### 2.7 Plasmid construction, dsRNAs design, and cell transfection

Full‐length cDNA of human TTP gene was amplified using reverse transcription‐polymerase PCR (RT‐PCR) from the total cDNA of UMUC3 and 5637 cells using special primers (Table S2). The amplified cDNA was then subcloned into the pcDNA3.1 vector for gene overexpression. The plasmid was transfected into bladder cells using Lipofectamine 3000 reagent (Invitrogen, USA) according to the manufacturer's instructions, then, the transfected cells were screened by G418 (InvivoGen, USA) at 48 hours after cell transfection. A 1 kb DNA promoter sequence of TTP was downloaded from Genome Browser (http://genome.ucsc.edu/). dsRNAs of TTP were designed by putting the promoter sequence into the Excel program referring to the previous step by step methods.^30^ And six dsRNAs were selected according to the program scores and synthesized by Ribobio company (Guangzhou, China), the sequences of the six dsRNA were listed in Table S3. Then, the dsRNAs were transfected into BCa cells using Lipofectamine RNAi MAX reagent (Invitrogen, USA) according to the manufacturer's instructions.

### 2.8 Protein extraction and western blotting

Total proteins of BCa cells were extracted using RIPA lysis buffer (Beyotime, China) according to the manufacturer's instructions, and BCA protein assay kit (Pierce, USA) was used to determine the protein concentrations. A 10 μg protein samples were fractionated using 10% sodium dodecyl sulfate polyacrylamide gel electrophoresis (SDS PAGE), and then, transferred to polyvinylidene fluoride (PVDF) membranes. Then, the protein binding PVDF membranes was blocked by incubating with 5% bovine serum albumin (BSA). The blocked membrane was incubated with primary antibodies including TTP (1/1000) (Abcam, UK), CDK1 (1/2000) (Abcam, UK), and GAPDH (1/2000) (Cell Signaling Technology, USA) at 4°C overnight. Finally, the membranes were incubated with corresponding secondary antibody (Cell Signaling Technology, USA) and detected by enhanced chemiluminescence assay kit (Millipore, USA).

### 2.9 Cell counting kit‐8 (CCK‐8) assay

BCa cells at the logarithmic phase were trypsinized and resuspended, and 2000 cells/wells were placed in 96 well plates. CCK8 (Yeason, China) was used to detected proliferation abilities of BCa cells by measuring OD values of 24, 48, and 72 hours at 450 nm using spectrophotometry (BioTek, USA).

### 2.10 Colony formation assay

The trypsinized cells were resuspended and seeded in 6‐well plates (1000 cells/wells) with complete medium. In order to maintain the cells growth, the medium was replaced every 3 days until 2 weeks. The colonies were then fixed with 100% methanol for 10 minutes and stained with 0.5% crystal violet (Beyotime, China) for 30 minutes. The staining colonies were photographed, and then, calculated using ImageJ.

### 2.11 Cell cycle assay

Transfected cells were harvested and fixed with 70% ethanol at 4 °C overnight. Then, the cells were stained with propidium iodide (PI, Beyotime, China) to conduct cell cycle analysis according to the manufacturer's protocol. Cell cycle distribution of each sample was analyzed in CytoFlex S (Beckman Coulter, USA) flow cytometry.

### 2.12 Wound healing assay

Transfected cells were trypsinized, and then, reseeded into 6‐well plates (10^6^ cells/wells). When the cell fusion is at 100%, the monolayer cells were scratched using 10 μl sterile pipette tips and the detached cells were washed off with sterilized PBS, then, 2 ml serum‐free medium was added into the wells. The scratch was photographed by the inverted microscope at 0, 24, and 48 hours (Olympus, Japan). Scratch width were measured using ImageJ for assessing cell migration abilities.

### 2.13 Cell invasion assay

24‐well Boyden chambers with 8 μm pore size polycarbonate membranes (Costar, USA) were precoated with the Matrigel (Corning, USA). For assessing abilities of cell invasion, 10^5^ cells were seeded into 24‐well Boyden chambers. Then, serum‐free medium was added into the upper chamber and medium with 20% FBS was added into the lower chamber for chemoattractant. After 24 hours, the nonmotile cells at the top of the membranes were removed with cotton swabs, and then, the membranes were fixed with 100% methanol for 10 minutes and stained with 0.5% crystal violet for 30 minutes. Staining cells of each membrane were randomly photographed using inverted microscope, and then, the numbers of staining cells were counted using ImageJ.

### 2.14 Luciferase reporter assay

Full‐length 3′ UTR of human CDK1 gene was amplified from the total cDNA of 5637 and UMUC3 cells using special primers (Table S2). Then, the amplified 3′ UTR cDNAs were subcloned into the psiCHECK2 Renilla/Firefly dual‐luciferase expression vector. psiCHECK2‐CDK1 3′ UTR plasmid was co‐transfected with pcDNA3.1 plasmid or pcDNA3.1/TTP plasmid using Lipofectamine 3000 reagent (Invitrogen, USA) according to the manufacturer's instructions. After 48 hours transfection, transfected cells were lysed with passive lysis buffer and mixed with luciferase assay reagent (Promega, USA) according to the manufacturer's instructions, and then, chemiluminescent signal was measured using luminometer (BioTek, USA). Renilla luciferase/Firefly luciferase of each sample was calculated to determine relative luciferase activity.

### 2.15 Tumor xenograft model

Female BALB/c nude mice at the age of 5 weeks were bought from Nanjing Biomedical Research Institute of Nanjing University and housed in a pathogen‐free animal facility (Research Center of Experimental Medicine, Ruijin Hospital) with free access to distilled water and food. After a week of acclimatization, 5 × 10^6^ UMUC3 cells were subcutaneously injected into the armpits of the nude mice. Chemically modified Control dsRNA and TTP dsRNA were synthesized for in vivo study by Ribobio company (Guangzhou, China). When the tumor volumes reached about 120 mm^3^, the mice were randomly divided into two groups (n = 6 per group), and then, Control dsRNA or TTP dsRNA dissolved in saline (5 nmol per mouse) was intratumorally injected into the tumor every 3 days. Tumor volumes were measured every 4 days according to the following formula: tumor volume = ½ × length × width^2^. All animal procedures were performed following approval from the Animal Care and Use Committee of Ruijin Hospital, and undertaken in accord with the National Institutes of Health Guide for the Care and Use of Laboratory Animals.

### 2.16 Statistical analysis

All data in this study were showed as the means ± standard deviation (MEAN±SD). Differences between two groups were compared using the Mann‐Whitney *U* test, with *p* value <0.05 considered statistically significant. Overall survivals (OS) of patients from two subgroups were compared using Kaplan‐Meier method following by log‐rank test.

## 3 RESULTS

### 3.1 TTP expression was downregulated in BCa

**Figure 1:**
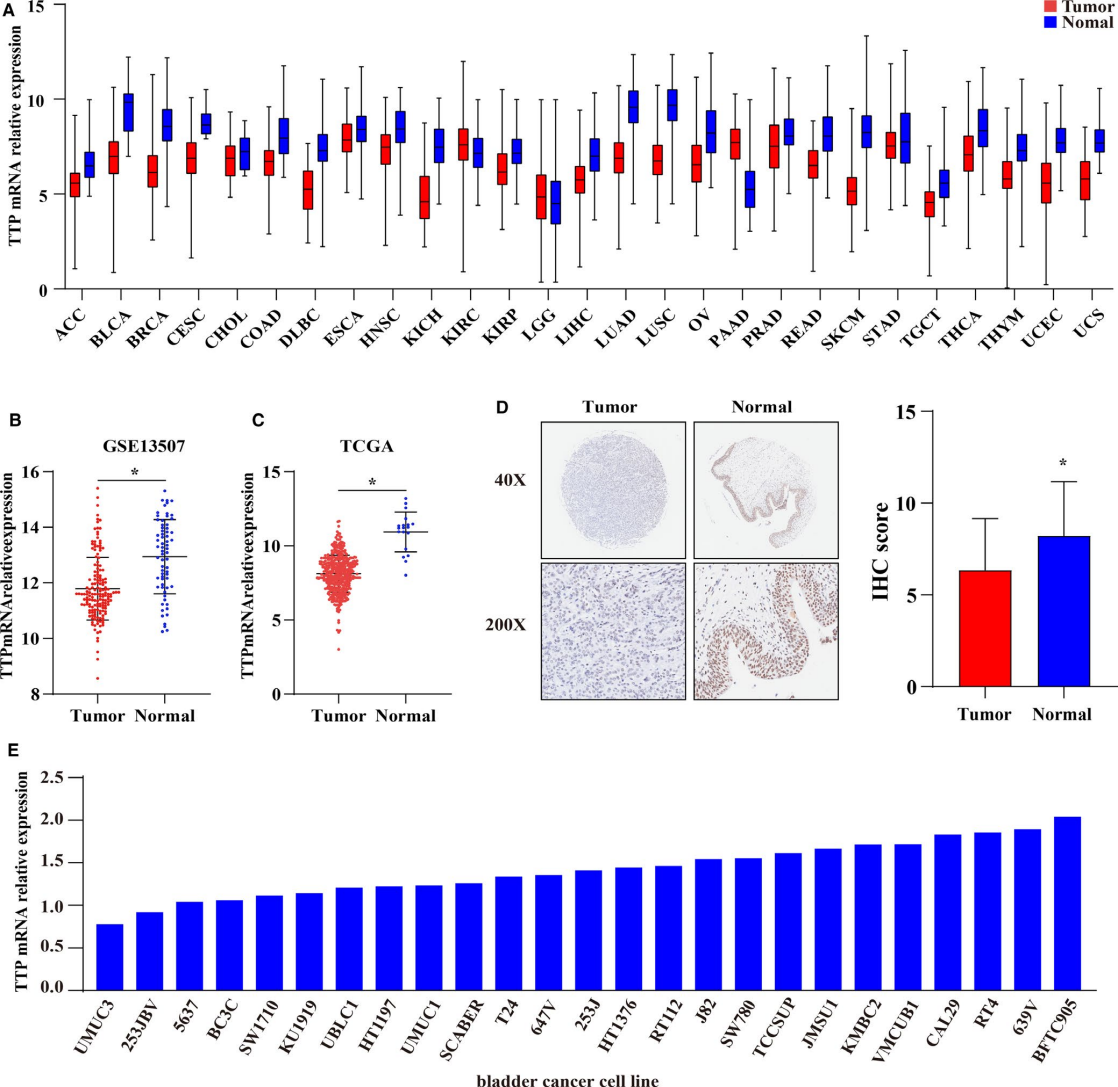
TTP expression was downregulated in BCa. (A) mRNA expression levels of TTP were downregulated in most tumors compared to the normal tissues (ACC, Adrenocortical carcinoma; BLCA, Bladder carcinoma; BRCA, Breast invasive carcinoma; CESC, Cervical squamous cell carcinoma and endocervical adenocarcinoma; CHOL, Cholangiocarcinoma; COAD; Colon adenocarcinoma; DLBC, Lymphoid neoplasm diffuse large B‐cell Lymphoma; ESCA, Esophageal carcinoma; KICH, Kidney chromophobe; KIRC, Kidney renal clear cell carcinoma; KIRP, Kidney renal papillary cell carcinoma; LGG, Lower grade glioma; LIHC, Liver hepatocellular carcinoma; LUAD, Lung adenocarcinoma; LUSC, Lung squamous cell carcinoma; OV, Ovarian serous cystadenocarcinoma; PAAD, Pancreatic adenocarcinoma; PRAD, Prostate adenocarcinoma; READ, Rectum adenocarcinoma; SKCM, Skin cutaneous melanoma; STAD, Stomach adenocarcinoma; TGCT, Testicular Germ Cell Tumors; THCA, Thyroid carcinoma; THYM, Thymoma; UCEC; Uterine corpus endometrial carcinoma; UCS, Uterine carcinosarcoma). mRNA expression levels of TTP decreased in the bladder tumors relative to the normal bladder epithelium: (B) GSE13507, 165 tumors and 67 normal tissues; (C) TCGA, 414 tumors and 19 normal tissues. (D) Tissue microarray analysis showed that the protein levels of TTP in bladder tumors (n = 59) were lower than those in normal tissues (n = 18). (E) mRNA expression levels of TTP in 25 common bladder cell lines from Cancer Cell Line Encyclopedia (CCLE). The differences between the two groups were compared by the Mann‐Whitney *U* test (*p < 0.05)

After comparing mRNA expression levels of TTP in 27 types of tumors and their corresponding normal tissues, we noticed that mRNA expression levels of TTP were downregulated in most tumors compared to the normal tissues (Figure 1A). Because expression levels of TTP in BCa had not been investigated until now, we then compared mRNA expressions of TTP in tumor samples and normal tissues from two BCa data sets: GSE13507 (165 tumors and 67 normal tissues) and TCGA‐BLCA (414 tumors and 19 normal tissues). We found that mRNA expression levels of TTP also decreased in the BCa relative to the normal bladder epithelium (Figure 1B,C). Protein levels of TTP in 59 bladder tumors and 18 normal tissues were then compared using tissue microarray, and the results validated that protein levels of TTP significantly decreased in the BCa compared to the bladder epithelium (Figure 1D). mRNA expression levels of TTP in 25 common bladder cell lines from CCLE were showed in Figure 1E, we found that mRNA expression levels of TTP in 5637, 253JBV, and UMUC3 cells were lowest and BFTC905, 639 V, and RT4 cells were highest among the 25 BCa cells.

### 3.2 Function enrichment analysis showed that TTP target genes were enriched in tumor‐associated pathways.

**Figure 2:**
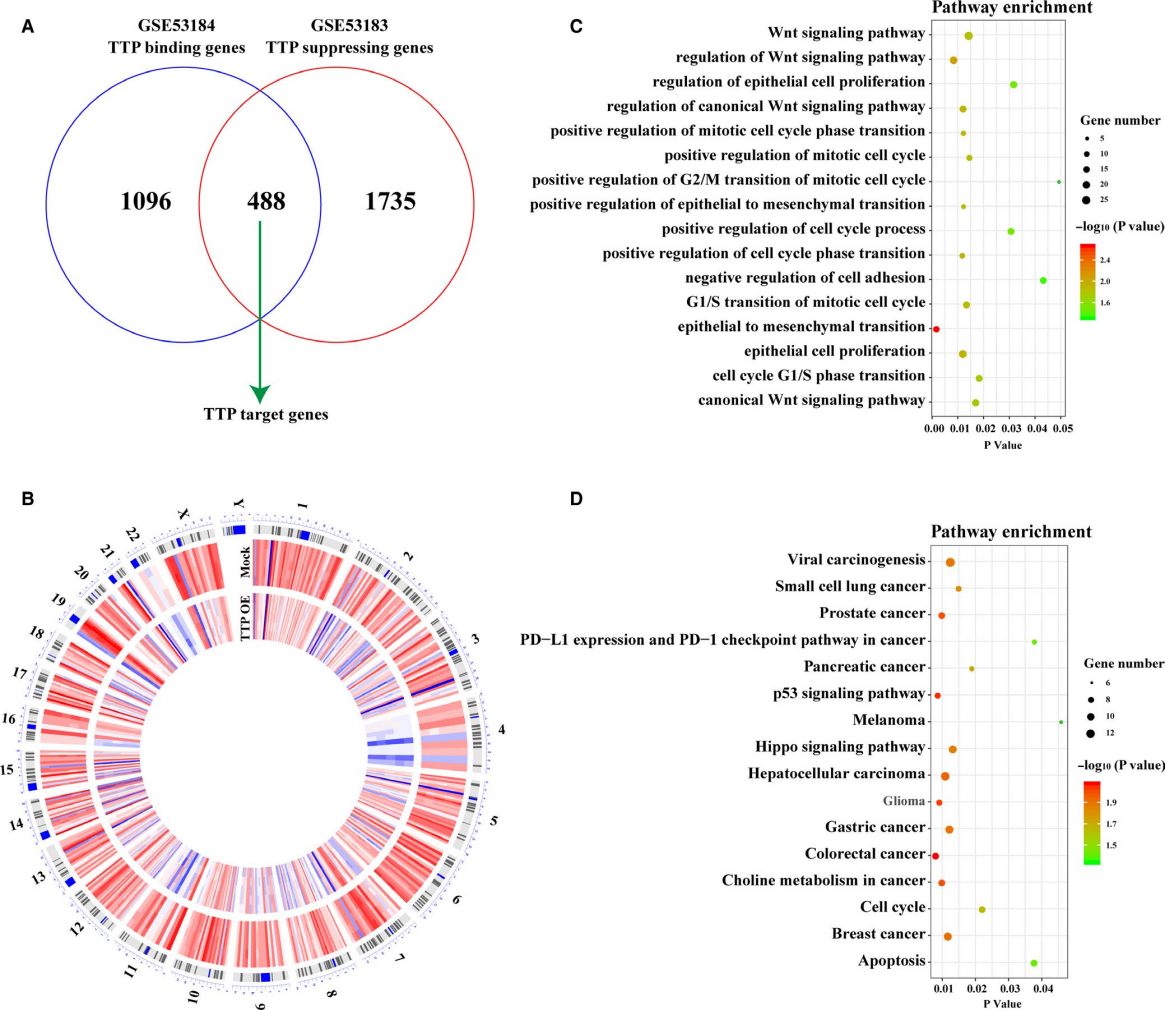
Function enrichment analysis showed that TTP target genes were enriched in tumor‐associated pathways. (A) mRNA expression levels of 2223 genes could be suppressed by TTP (GSE53183), 1584 genes were found to have TTP binding sites in their 3′ UTRs (GSE53184), and a total of 488 TTP target genes which could be both suppressed and bound by TTP were finally identified. (B) The 488 genes were distributed on the 22 pairs of human chromosomes. (C) The 488 TTP target genes were enriched in several significant Gene Ontology (GO) terms such as positive regulation of mitotic cell cycle, epithelial to mesenchymal transition (EMT), and Wnt signaling pathway. (D) The 488 TTP target genes were enriched in several Kyoto Encyclopedia of Genes and Genomes (KEGG) pathways such as viral carcinogenesis, PD‐L1 expression and PD‐1 checkpoint pathway in cancer, and cell cycle

As previous studies reported, TTP could suppress mRNA expressions of downstream target genes by binding AREs in their 3′ UTRs.^6^, ^22^, ^23^, ^24^ Then, we got a list of TTP target genes via comprehensively analyzing public TTP overexpression microarray data (GSE53183) and PAR‐CLIP sequencing data (GSE53184). As shown in Figure 2A, mRNA expression levels of 2223 genes were found to be suppressed by TTP(GSE53183), 1584 genes were found to have TTP binding sites in their 3′ UTRs (GSE53184), and a total of 488 TTP target genes which could be both suppressed and bound by TTP were finally identified. As shown in Figure 2B, the 488 genes were distributed on the 22 pairs of human chromosomes. Then, function enrichment analysis was performed to explore biological functions of TPP target genes, and several significant Go terms and KEGG pathways were enriched. For Go analysis, the enriched Go terms included positive regulation of mitotic cell cycle, epithelial to mesenchymal transition (EMT), and Wnt signaling pathway (Figure 2C). For KEGG pathways analysis, several tumor‐associated pathways such as viral carcinogenesis, PD‐L1 expression and PD‐1 checkpoint pathway in cancer, and cell cycle were enriched (Figure 2D).

### 3.3 High expressions of TTP target genes in BCa were associated with worse clinical outcome and stronger activities of cell cycle associated pathways.

**Figure 3:**
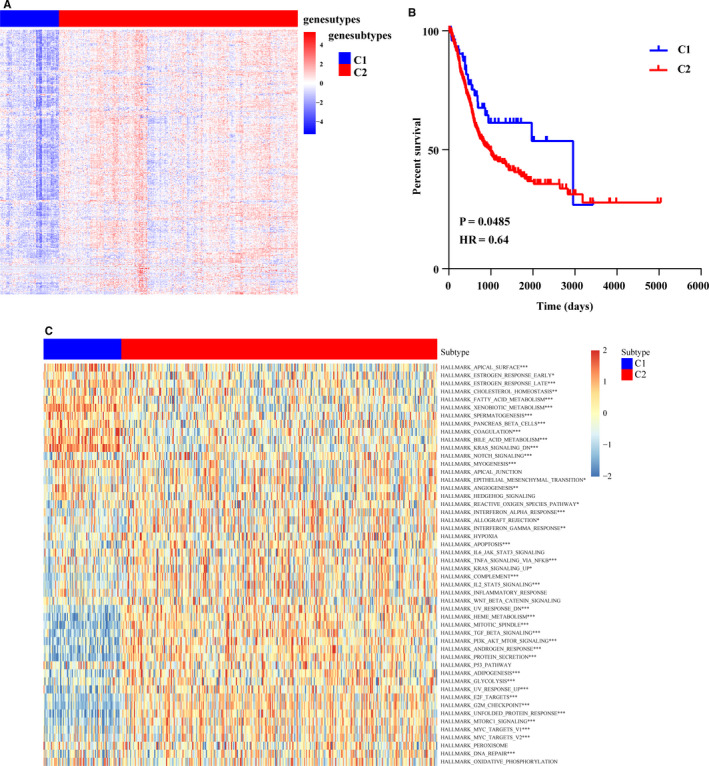
High expressions of TTP target genes in BCa were associated with worse clinical outcome and stronger activities of cell cycle associated pathways. (A) 405 patients from TCGA cohort were divided into two subtypes (C1 (n = 80) and C2 (n = 325)) according to the mRNA expression levels of TTP target genes, and the mRNA expression levels of most TTP target genes were higher in C1 subtype than those in C2 subtype. (B) Overall survival (OS) of C1 subtype patients was significantly longer than that of C2 subtype patients (Log‐rank test, *p* = 0.0485). (C) Enrichment scores of pathways such as E2F target, G2 M checkpoint, MYC target, MTORC1 signaling, PI3 K‐AKT‐MTOR signaling, and mitotic spindle were significantly lower in patients of C1 subtype than those in patients of C2 subtype. The differences between the two groups were compared by the Mann‐Whitney *U* test (*p < 0.05)

In order to further explore biological roles of TTP target genes in BCa, we exacted expression matrix of TTP target genes from the RNA‐seq matrix of 405 patients with BCa from TCGA‐BLCA cohort to perform cluster analysis. As showed in Figure 3A, 405 patients could be divided into two subtypes: C1 (n = 80) and C2 (n = 325), and mRNA expression levels of most TTP target genes were higher in C2 subtype than that in C1 subtypes. Then, overall survival (OS) of C1 subtype patients and that of C2 subtype patients were compared, the results showed that OS of C1 subtype was significantly longer than that of C2 subtype (Figure 3B, *p* < 0.05). Then, fisher's exact test was employed to explore relationships between the subtypes and the clinicopathological characteristics of BCa patients. We found that patients in C1 subtype had better survival status (*p* = 0.001), lower rates of node metastasis (*p* = 0.014), lower TNM stage (*p* = 0.003), and lower pathological grade (*p* < 0.05) (Table 1). In order to explore biological pathway differences between the two subgroups, enrichment scores of 50 hallmark pathways in 405 patients from the two groups were generated, and then, compared. We found that enrichment scores of pathways such as E2F target, G2 M checkpoint, MYC target, MTORC1 signaling, PI3K‐AKT‐MTOR signaling, and mitotic spindle were obviously lower in patients of C1 subtype than those in patients of C2 subtype (Figure 3C). In total, the above results indicated that BCa patients with higher expression levels of TTP target genes had worse clinical outcome and stronger activities of cell proliferation, so we speculated that TTP might be able to suppress aggressiveness of BCa by inhibiting expression of its target genes like other tumors.

**Table 1:** Clinicopathological characteristics of patients from two BCa subtypes

Factors	subtypes		*p* value^a^
	C1	C2	
Age			
<60	23 (26.74%)	63 (73.26%)	0.092
>=60	57 (17.87%)	262 (82.13%)	
Gender			
Male	57 (71.25%)	242 (74.46%)	0.571
Female	23 (28.75%)	83 (25.54%)	
Status			
Alive	57 (72.15%)	169 (52.16%)	0.001
Dead	22 (27.85%)	155 (47.84%)	
T stage			
T1+T2	45 (56.96%)	157 (48.91%)	0.211
T3+T4	34 (43.04%)	164 (51.09%)	
N stage			
N0	57 (77.03%)	178 (61.59%)	0.014
N1+N2+N3	17 (22.97%)	111 (38.41%)	
TNM stage			
I+II	38 (47.50%)	95 (29.32%)	0.003
III+IV	42 (52.50%)	229 (70.68%)	
Grade			
Low grade	15 (18.75%)	6 (1.86%)	<0.05
High grade	65 (81.25%)	316 (98.14%)	

^a^ *p* values are from Fisher's exact test and were statistically significant at <0.05.

### 3.4 TTP suppressed proliferation, migration, and invasion of BCa cells.

**Figure 4:**
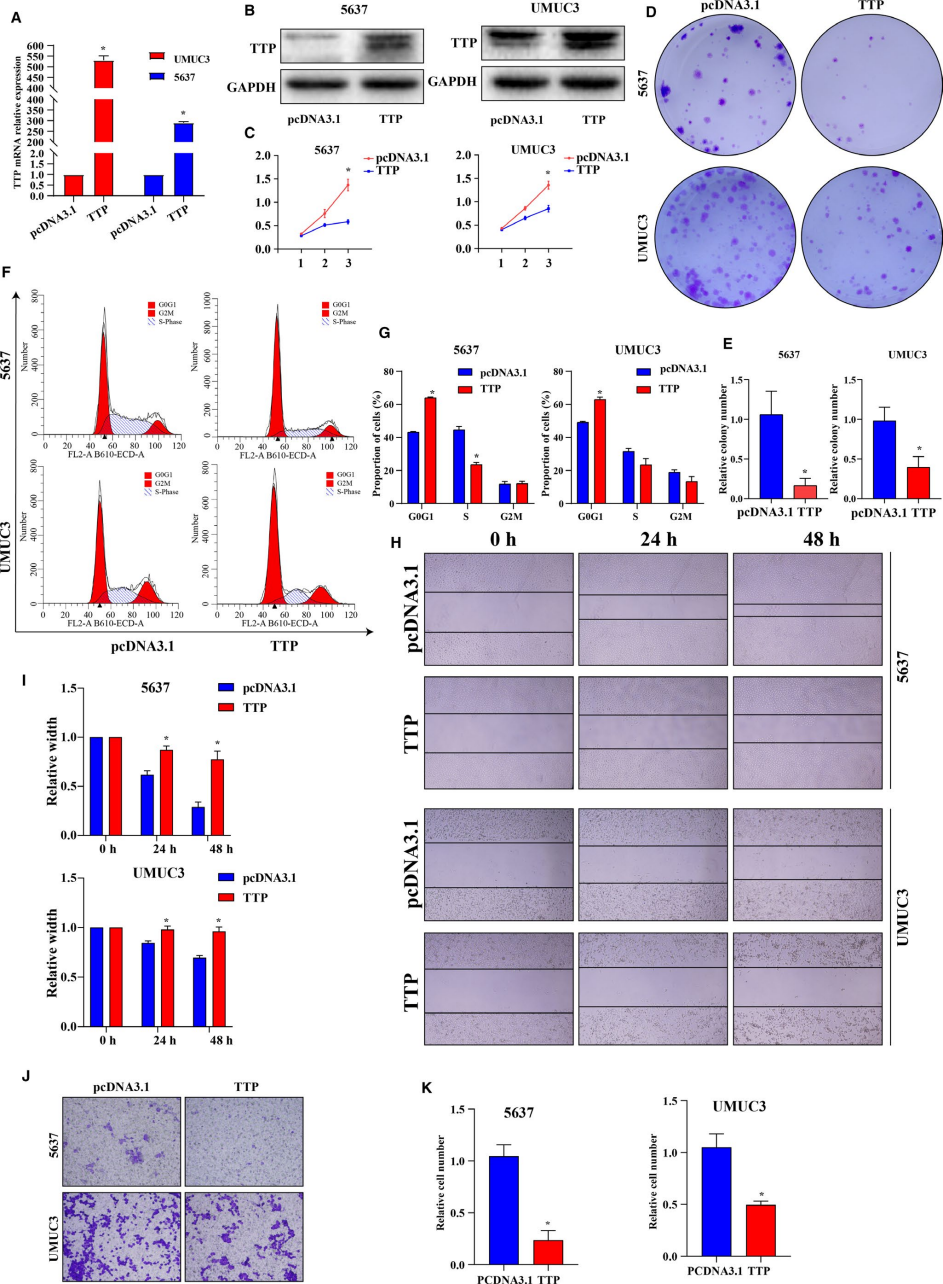
TTP suppressed proliferation, migration, and invasion of bladder cancer. (A) mRNA and (B) protein levels of TTP in 5637 and UMUC3 cells were detected by Quantitative Real‐time PCR (qRT‐PCR) and western‐blot after transfecting pcDNA3.1/TTP plasmid, respectively. (C) CCK8 assay showed that TTP could suppress viabilities of 5637 and UMUC3 cells. (D and E) Colony formation assay showed that colony formation abilities of 5637 and UMUC3 cells weakened after TTP transfection. (F and G) Cell cycle assay showed that TTP could induce BCa cell cycle arresting at the G0/G1 phase. (H and I) Wound healing assay showed TTP could suppress cell migration of 5637 and UMUC3 cells. (J and K) Transwell assay showed that TTP could suppress invasion of 5637 and UMUC3 cells. The differences between the two groups were compared by the Mann‐Whitney *U* test (*p < 0.05)

Because biological functions of TTP have not been explored in BCa until now, our bioinformatics analysis suggested that TTP might be a tumor suppressor which was downregulated in BCa. In order to validate the analysis results, TTP overexpression 5637 and UMUC3 cells were constructed by transfecting pcDNA3.1/TTP plasmid (Figure 4A,B). CCK8 and colony formation assays showed that TTP overexpression could suppress cell viabilities (Figure 4C, all *p* < 0.05) and proliferation (Figure 4D,E, all *p* < 0.05) of 5637 and UMUC3 cells. Cell cycle assay showed that TTP overexpression could induce BCa cell cycle arresting at the G0/G1 phase (Figure 4F,G, all *p* < 0.05). Then, cell migration and invasion abilities were evaluated using wound healing assay and transwell assay, respectively, and the results showed that TTP overexpression could suppress migration (Figure 4H,I, all *p* < 0.05) and invasion abilities (Figure 4J,K, all *p* < 0.05) of 5637 and UMUC3 cells. In total, these in vitro experiments validated that TTP could inhibit aggressiveness of BCa cells.

### 3.5 TTP suppressed expression of cycle dependent kinase 1 (CDK1) via targeting its 3′ UTR.

**Figure 5:**
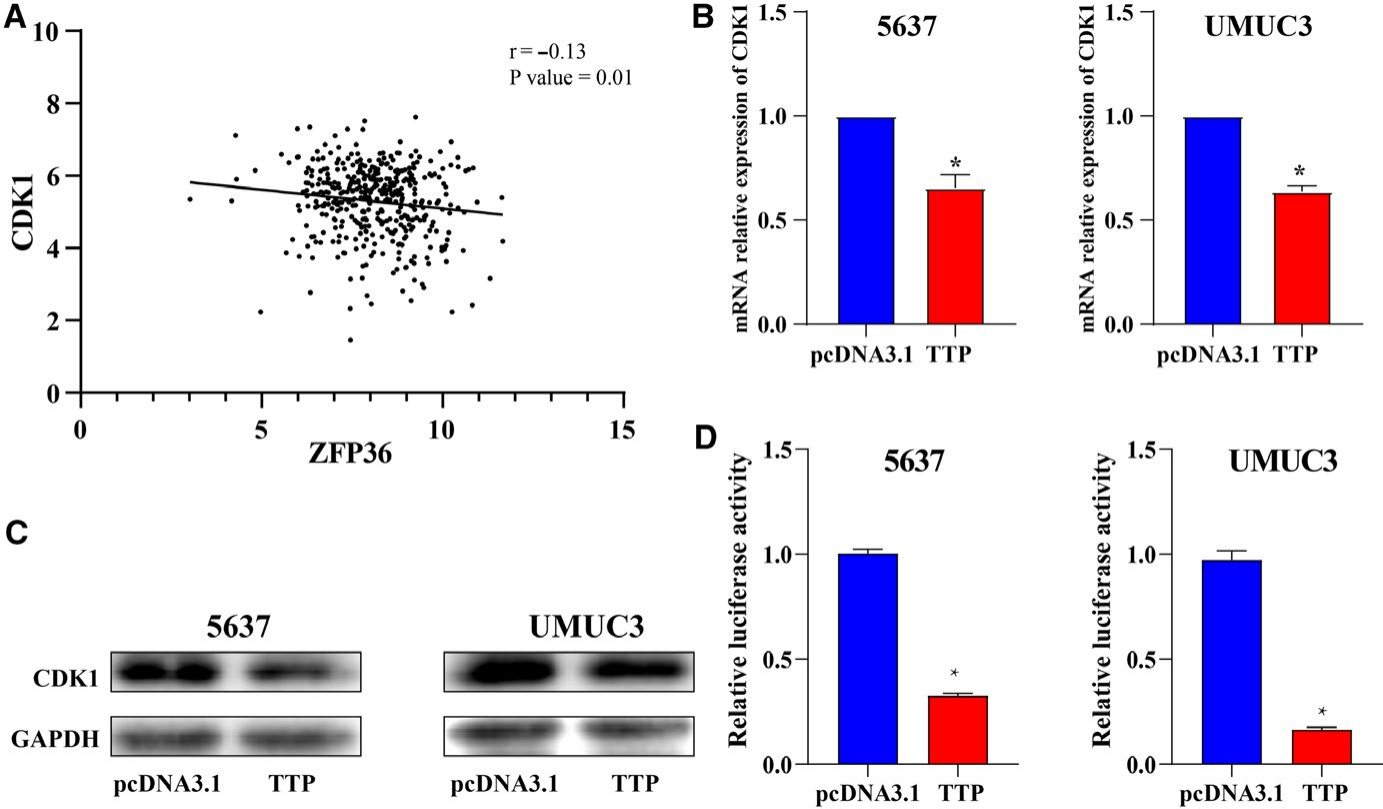
TTP suppressed expression of cycle dependent kinase 1 (CDK1) via targeting its 3′ UTRs. (A) Correlation analysis indicated that CDK1 was negatively correlated with TTP in TCGA‐BLCA cohort (*p* = 0.01). (B) mRNA and (C) protein expression levels of CDK1 in TTP overexpression 5637 and UMUC3 cells were lower than those in control cells (D) Luciferase reporter assay indicated that that TTP could directly targeted CDK1 by binding its 3′ UTR. The differences between the two groups were compared by the Mann‐Whitney *U* test (*p < 0.05)

As an RNA binding protein, mRNAs of several proto‐oncogene had been experimentally demonstrated to be directly inhibited by TTP.^9^, ^13^, ^15^, ^31^ To explore whether other genes were directly regulated by TTP, then, we screened potential genes in a Go term named positive regulation of cell cycle phase transition from the enrichment analysis results. This Go term included nine genes: CUL3, RDX, AKT1, RAB11A, CDK1, ADAMTS1, MDM2, CDC73, RCC2. Correlation analysis was performed to explore relationships between TTP and the nine genes using RNA‐seq data from TCGA, and the results indicated that CDK1 was negatively correlated with TTP (Figure 5A, *p* = 0.01). Therefore, we speculated that CDK1 was most likely to be directly regulated by TTP among the nine genes. Then, we detected mRNA and protein expressions in TTP overexpression cells to validate whether CDK1 could be regulated by TTP, and excitedly found that TTP overexpression could decrease expression levels of CDK1 in 5637 and UMUC3 cells (Figure 5B,C). Luciferase reporter assay indicated that that TTP could directly target CDK1 by binding its 3′ UTR (Figure 5D).

### 3.6 Designing and screening of dsRNAs for inducing TTP expression in BCa.

**Figure 6:**
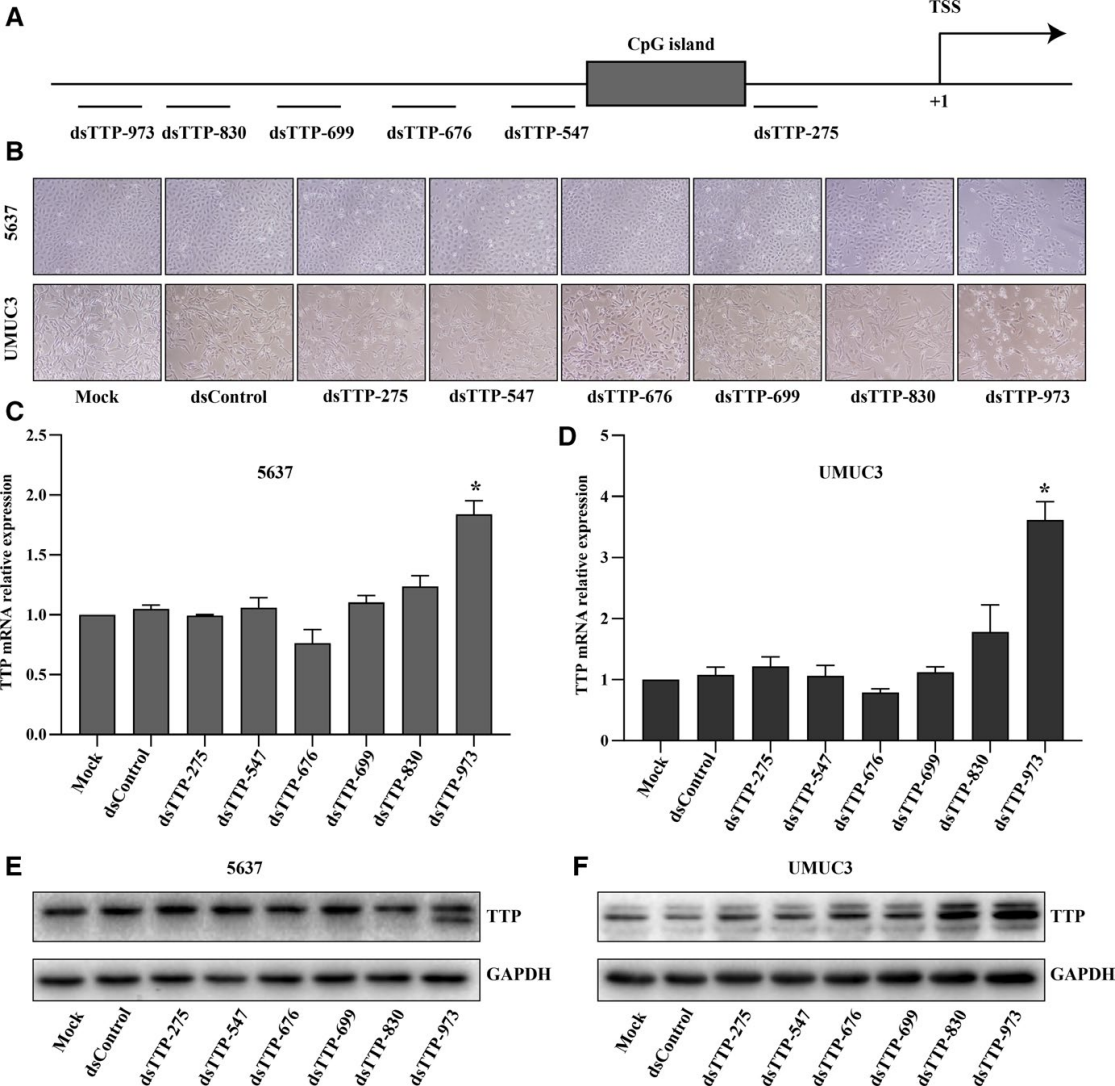
Design and screening of dsRNAs for inducing TTP expression in BCa. (A) The six dsRNAs were distributed on 1 kb promoter region upstream of transcript start site (TSS) of the TTP gene. (B) Cell morphology differences of 5637 and UMUC3 cells were observed under a microscope after 72 h transfection of the six dsRNAs. (C and D) dsTTP‐973 could induce TTP mRNA expression in 5637 and UMUC3 cells among the six dsRNAs (Comparing with dsControl group, *p* < 0.05). (E and F) dsTTP‐973 could successfully induce TTP protein expression in 5637 and UMUC3 cells among the six dsRNAs (*p < 0.05)

As we found above, TTP expression decreased in BCa relative to the normal bladder tissue and TTP overexpression could suppress aggressiveness of BCa. Therefore, induction of TTP expression might be a potential therapeutic strategy for BCa. dsRNAs are newly discovered gene activation tools and have been shown to activate the expression of multiple tumor suppressor genes in tumor cells.^19^, ^32^, ^33^, ^34^ Referring to the previous designs of dsRNAs,^30^ we designed and selected six candidate dsRNAs (dsTTP‐275, dsTTP‐547, dsTTP‐676, dsTTP‐699, dsTTP‐830, and dsTTP‐973) targeting promoter of TTP according to the quality scores of dsRNAs (Table 2 and Table S3). As shown in Figure 6A, the six dsRNAs were distributed on 1 kb promoter region upstream of transcript start site (TSS) of the TTP gene. The six dsRNAs were synthesized artificially, and then, transfected into 5637 and UMUC3 cells, and cell morphology was observed under a microscope after 72 h transfection. We found that cells in dsTTP‐973 group appeared significant atrophy compared to cells in other groups (Figure 6B). To verify whether the TTP expression was elevated after dsRNA transfection, mRNA and protein levels of dsRNAs transfected cells were detected by PCR and western‐blot, respectively. And we found that only dsTTP‐973 successfully induce TTP expression in 5637 and UMUC3 cells at mRNA (Figure 6C,D, all *p* < 0.05 comparing with dsControl group) and protein level (Figure 6E,F) among the six dsRNAs.

**Table 2:** Location and scoring information of the six dsRNAs targeting TTP promoter

Name	Range	GC%	Position from TSS	Score
dsTTP‐275	275‐293	47.4	275	5
dsTTP‐547	547‐565	42.1	547	5.5
dsTTP‐676	676‐694	52.6	676	6
dsTTP‐699	699‐717	52.6	699	5.5
dsTTP‐830	830‐848	52.6	830	5.5
dsTTP‐973	973‐991	47.4	973	5.5

### 3.7 dsRNA‐973 suppressed proliferation, migration, and invasion of BCa in vitro.

**Figure 7:**
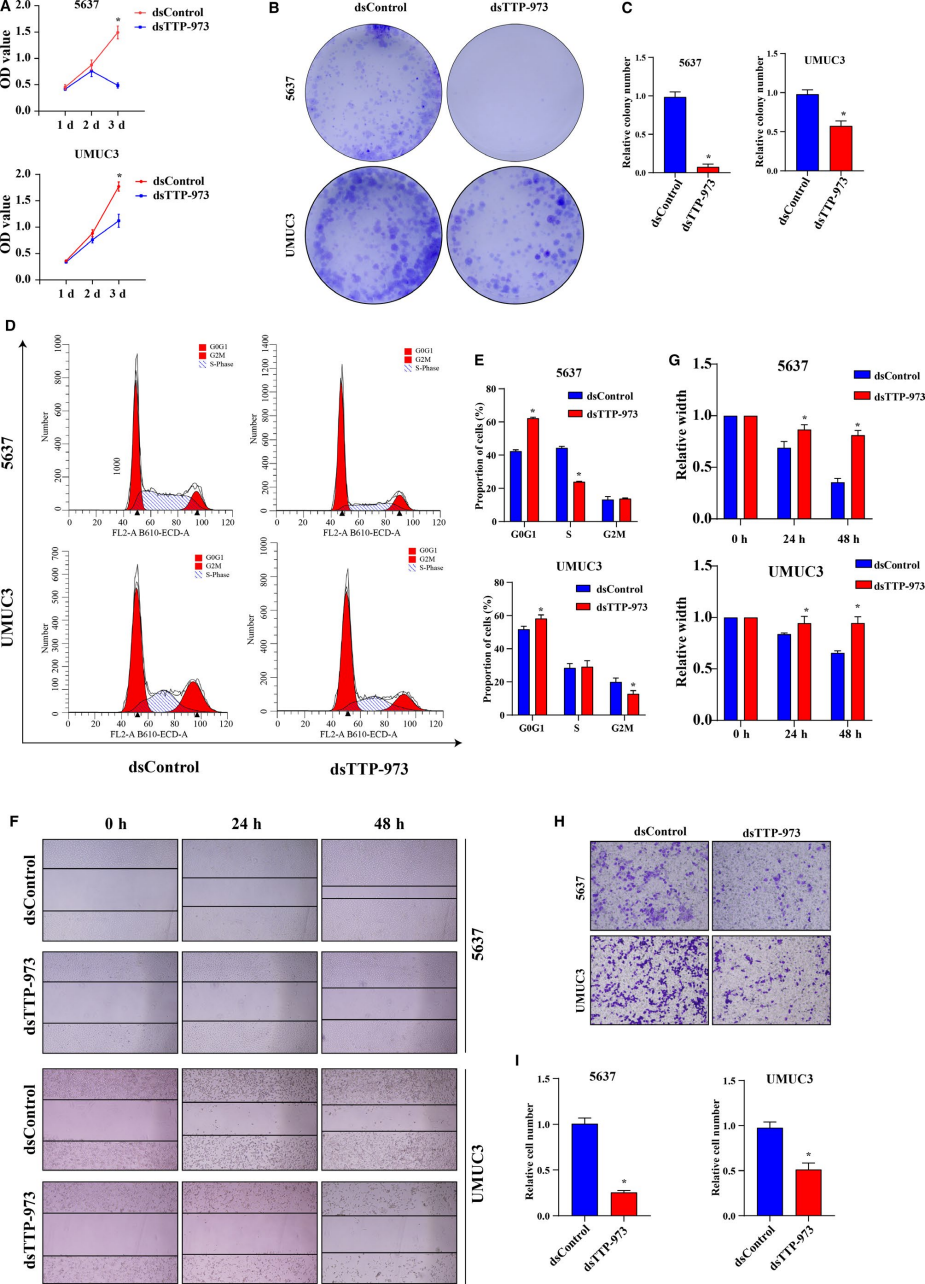
dsRNA‐973 suppressed proliferation, migration, and invasion of BCa in vitro. (A) CCK8 assay showed that cell viabilities of 5637 and UMUC3 cells decreased after dsTTP‐973 transfection. (B and C) Colony formation assay showed that colony formation abilities of 5637 and UMUC3 cells weakened after dsTTP‐973 transfection. (D and E) Cell cycle assay showed that dsTTP‐973 could induce 5637 and UMUC3 cell cycle arresting at the G0/G1 phase. (F and G) Wound healing assay showed that cell migration of 5637 and UMUC3 cells became slower after dsTTP‐973 transfection. (H and I) Transwell assay showed that invasion abilities of 5637 and UMUC3 cells weakened after dsTTP‐973 transfection. The differences between the two groups were compared by the Mann‐Whitney *U* test (* *p* < 0.05)

Then, we performed in vitro experiments to further determine whether dsTTP‐973 could inhibit aggressiveness of BCa cells. Similar to the TTP overexpression, cell viabilities of 5637 and UMUC3 cells significantly decreased after 72 hours dsTTP‐973 transfection (Figure 7A, all *p* value<0.05), and colony formational ability of BCa also weakened after dsTTP‐973 transfection (Figure 7B,C, all *p* < 0.05). Flow cytometry analysis showed that cell proportions of dsTTP‐973 transfected cells in the G0/G1 phase were higher comparing with the dsControl transfected cells (Figure 7D,E, all *p* < 0.05), the result indicated that dsTTP‐973 could induce cell cycle arrest of BCa cells. Wound healing assay showed that cell migration of 5637 and UMUC3 became slower after dsTTP‐973 transfection at 24 and 48 hours (Figure 7F,G, all *p* < 0.05), and transwell assay revealed that invasion abilities of 5637 and UMUC3 cells weakened after dsTTP‐973 transfection (Figure 7H,I, all *p* < 0.05).

### 3.8 dsRNA‐973 suppressed the growth of BCa cells in vivo.

**Figure 8:**
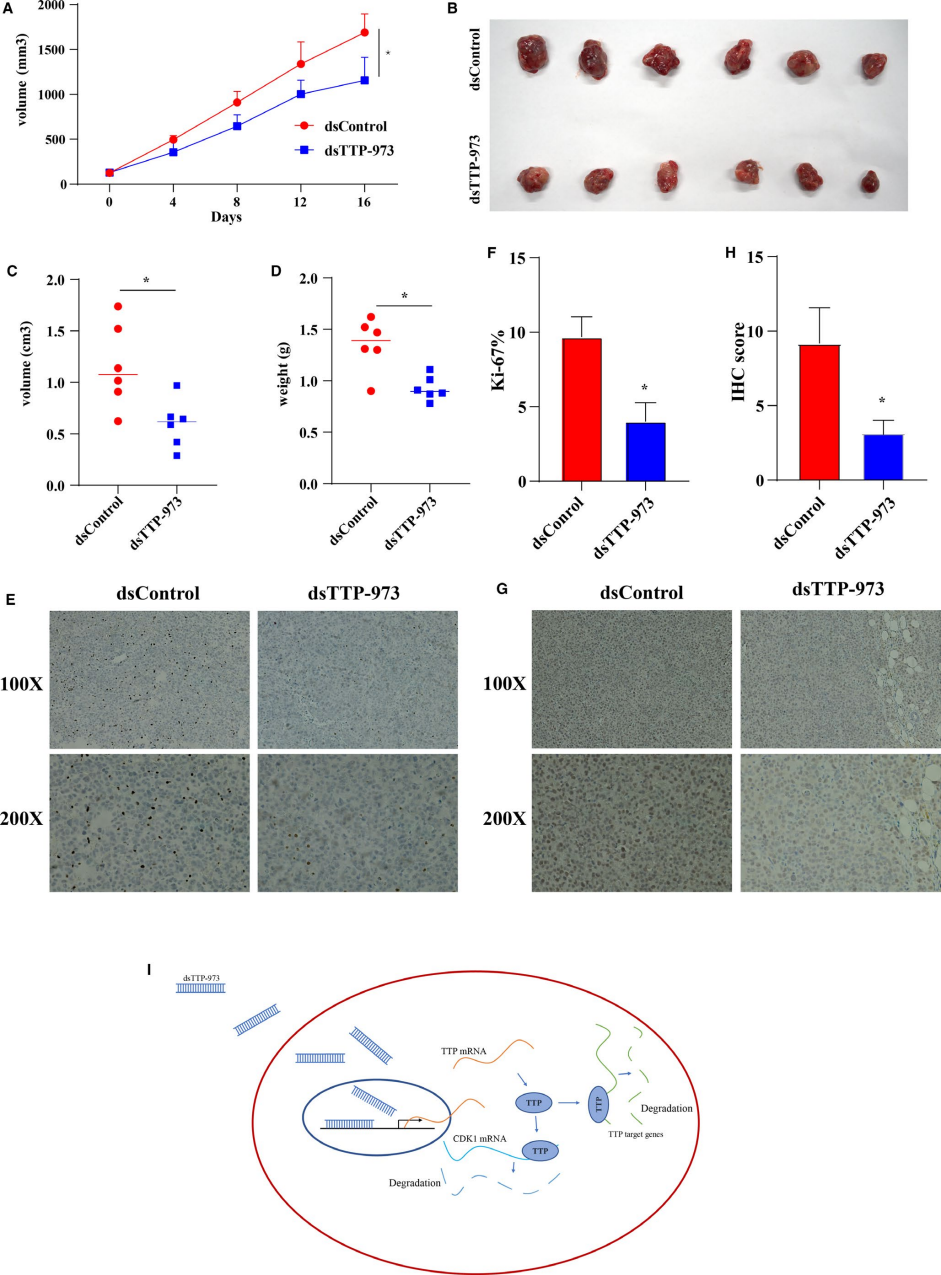
dsRNA‐973 suppressed the growth of BCa cells in vivo. (A) After six continuously injections, growth rates of bladder tumors in the mice injected with dsTTP‐973 were slower than those in the mice injected with dsControl. (B and C) Tumor sizes and (D) weights in dsTTP‐973 group significantly reduced compared with those in dsControl group. Representative images showing immunohistochemical staining of (E) Ki67 and (G) PCNA on tumors of two groups are shown. IHC analysis showed that the protein levels of (F) Ki‐67 and (H) PCNA in dsTTP‐973 group were lower than those in dsControl group. (I) Summary of the antitumor signaling of dsRNA‐973 in BCa. The differences between the two groups were compared by the Mann‐Whitney *U* test (*p < 0.05)

To validated antitumor effect of dsTTP‐973 in vivo, cholesterol‐modified dsTTP‐973 was intratumorally injected into the tumors of nude mice. After six continuously injections, we found that growth rates of tumors in mice injected with dsTTP‐973 were slower than those in mice injected with dsControl (Figure 8A, *p* < 0.05). Meanwhile, tumor sizes (Figure 8B,C, *p* < 0.05) and weights (Figure 8D, *p* < 0.05) in dsTTP‐973 group significantly reduced compared with those in dsControl group. Then, the cell proliferation associated makers including ki67 and PCNA were stained, and we found that densities of ki67 (Figure 8E,H) and PCNA (Figure 8G,H) were also lower in tumors of dsTTP‐973 groups than those in tumors of dsControl groups. Summary of the antitumor signaling of dsRNA‐973 in BCa cells was shown in Figure 8I. The above in vitro and in vivo experiments indicated that dsTTP‐973 had promising therapeutic effect for BCa.

## 4 DISCUSSION

As an RNA‑binding protein, TTP could destabilize mRNAs of target genes via binding AREs in their 3′ UTRs.^6^, ^7^, ^22^, ^35^ Several studies have reported that mRNAs of several oncogenes have AREs on their 3′ UTRs for TTP binding and abnormal posttranscriptional regulation mediated by TTP can induce tumor progression.^36^, ^37^ Edward et al^35^ found that mRNAs of 11 ARE genes (CDC6, KIF11, PRC1, NEK2, NCAPG, CENPA, NUF2, KIF18A, CENPE, PBK, and TOP2A) were overexpressed in tumors and negatively correlated with TTP/HuR mRNA ratios, function enrichment analysis showed that these genes were all associated with the mitotic cell cycle. In this study, we identified 488 TTP target genes which could both be suppressed and bound by TTP, function enrichment analysis showed that some of these genes were also associated with cell cycle‐related pathways. Because biological functions of TTP have not been investigated until now, we then explored expressions of the TTP target genes in BCa patients. BCa patients were then divided into two subtypes according to the mRNA abundances of TTP target genes in tumors of 405 patients from TCGA‐BLCA cohort, and we found that patients with high expression of TTP target genes had worse survival outcome, higher pathological stage, and stronger activities of proliferation associating pathways than patients with low expression of these genes. As an upstream regulatory gene, these results indicated that TTP might act as also an anticancer gene in BCa by interacting with oncogene mRNAs, and in vitro experiments validated that TTP could indeed suppress proliferation, migration, and invasion of BCa cells.

Transcriptome data and tissue microarray analysis validated that expression levels of TTP in BCa were lower than those in bladder epithelium. TTP has also been reported to be downregulated in several other tumors such as glioma,^8^ colon cancer,^9^ gastric cancer,^10^ and liver cancer,^11^ and several studies have explored TTP expression regulation mechanism in tumor. First at the transcriptional level, TTP expression could be directly induced by transcription factor ELK‐1 and EGR‐1 through binding the TTP promoter^38^; Methylation of a specific single CpG site (−500 bp) within the TTP promoter region could suppress TTP transcription in HCC cell and blockade of DNA methylation in this CpG site could increase TTP expression.^11^ Then, at the posttranscriptional level, miR‐29a could bind TTP 3′ UTR to suppress TTP expression, and overexpressed miR‐29a in pancreatic cancer could cause the expression silence of TTP.^39^ Finally, at the protein level, TTP protein could be phosphorylated and inactivated by MK2, which could be phosphorylates and activates by p38 kinase.^40^ Therefore, the mechanisms of TTP expression regulation are very complex, and understanding the specific mechanism will be helpful for the future antitumor therapy.

By screening genes from a Go terms named positive regulation of cell cycle phase transition, we inferred that CDK1 3′ UTR might be potentially targeted by TTP in BCa. Through luciferase assay, we validated that CDK1 was indeed downregulated by TTP in a 3′ UTR binding manner. CDK1 is a crucial cell cycle impeller and can promote cell G2/M transition via forming complex with cyclin B1.^41^ CDK1 has been reported to be upregulated in multiple type tumors such as diffuse large B cell lymphomas and melanomas.^42^ Meanwhile, CDK1 is an important antitumor target for inhibitor development in recent years, so it is very important to understand the regulation mechanisms of CDK1 expression for tumor therapy.^42^ Several studies had revealed that CDK1 could be regulated at the posttranscriptional level. For example, miRNA‐490‐3p,^43^ miR‐31,^44^ and miR‐186^45^ could inhibit tumor progression by degrading CDK1 mRNA by targeting its 3′ UTR. CDK1 mRNA could also been stabilized in an m6A‐independent manner by KIAA1429 which could promote breast cancer.^46^ Our study reveals a novel CDK1 regulatory mechanism which is dependent on TTP, and this finding can help us better understand the regulation mechanism of CDK1.

Similar to other cancers, our study validates that TTP is also a cancer suppressor gene in BCa. Therefore, we speculated that reactivation of TTP could be a promising strategy for treatment of BCa. In this study, we designed six dsRNAs to induce expression of TTP in BCa and found that dsTTP‐973 could significantly increase expression of TTP and suppress aggressiveness of BCa in vitro and in vivo. This encouraging result suggested dsTTP‐973 has potential for future clinical applications. However, we noticed that there are several problems need to solve before dsTTP‐973 use. First, in this study, the activation efficiencies were different in the two BCa cells, we thought that this phenomenon might be caused by epigenetic heterogeneities between different cell lines. Previous study indicated that promoter CpG islands and histone methylation status could affect dsRNAs responsiveness, and it suggested that DNA demethylation agents and histone deacetylase inhibitors are needed to promote susceptibility to dsRNAs in some situations.^30^ Another problem is dsRNAs delivery in vivo. With the development of material, several delivery tools such as lipid‐based system, dendrimer, lipopolyplex, and aptamer have been developed, and some of these have been already in clinical trials.^47^

## 5 CONCLUSIONS

In conclusion, TTP overexpression can inhibit the malignant biological behaviors of BCa, and can be a potential therapeutic target for BCa. dsTTP‐973 which can induce TTP expression in BCa is able to suppress BCa progression, and this dsRNA may be a potential treatment for BCa in the future.

## CONFLICTS OF INTEREST

The authors declare no conflicts of interest.

## AUTHOR CONTRIBUTIONS

WJ, CHW, and YZ conceived and designed this study; WJ performed the experiments and analysis procedures; WJ and DDZ contributed to the writing of the manuscript; WJ, DDZ, CHW, and YZ contributed to the revision of the manuscript. All authors read and approved the final manuscript.

## Supporting information

Table S1

Table S2

Table S3

## Data Availability

The data sets supporting the conclusions of this article are included within the article.
