# Supplementary material for: Tumor suppressing effects of tristetraprolin and its small double‐stranded RNAs in bladder cancer
Source: Cancer Med. 2020 Dec 1;10(1):269–85. doi: 10.1002/cam4.3622 (PMC7826468; doi:10.1002/cam4.3622)
Supplement: Supplementary file 1 — Table S1 [file CAM4-10-269-s001.docx]

**Table S1. Sequences for real time quantitative PCR primers used in present study.**

| **Primer name** | **Sequences** |
| --- | --- |
| TTP-F | CTCGCGCTACAAGACTGAGCTA |
| TTP-R | GGTCTTCGCTAGGGTTGTGGAT |
| CDK1-F | CTGGCTCTTGGAAATTGAGCGG |
| CDK1-R | AATGGGTATGGTAGATCCCGGC |
| GAPDH-F | CATGAGAAGTATGACAACAGCCT |
| GAPDH-R | AGTCCTTCCACGATACCAAAGT |
