# Supplementary material for: Tumor suppressing effects of tristetraprolin and its small double‐stranded RNAs in bladder cancer
Source: Cancer Med. 2020 Dec 1;10(1):269–85. doi: 10.1002/cam4.3622 (PMC7826468; doi:10.1002/cam4.3622)
Supplement: Supplementary file 2 — Table S2 [file CAM4-10-269-s002.docx]

**Table S2. Sequences for molecular cloning PCR primers used in present study.**

| **Primer name** | **Sequences** |
| --- | --- |
| TTP-F | ACCGTTACACCATGGATCTGAC |
| TTP-R | GTCACTCAGAAACAGAGATGCGATTG |
| CDK1-3’-UTR-F | TCAGATTAAGAAGATGTAGCTTTCTGAC |
| CDK1-3’-UTR -R | TAGATGGCTGCTAATAAACACTATGTC |
