# Supplementary material for: Tumor suppressing effects of tristetraprolin and its small double‐stranded RNAs in bladder cancer
Source: Cancer Med. 2020 Dec 1;10(1):269–85. doi: 10.1002/cam4.3622 (PMC7826468; doi:10.1002/cam4.3622)
Supplement: Supplementary file 3 — Table S3 [file CAM4-10-269-s003.docx]

**Table S3. Sequences for dsRNAs used in present study.**

| **Primer name** | **Sequences** |
| --- | --- |
| dsTTP-275 S | CUCAGUCUCCAGCUUUGAA[dT][dT] |
| dsTTP-275 AS | UUCAAAGCUGGAGACUGAG[dT][dT] |
| dsTTP-547 S | ACCCUCUUCUCCCUCUGAA[dT][dT] |
| dsTTP-547 AS | UUCAGAGGGAGAAGAGGGU[dT][dT] |
| dsTTP-676 S | GGGAAACCCCGUCUUUCAA[dT][dT] |
| dsTTP-676 AS | UUGAAAGACGGGGUUUCCC[dT][dT] |
| dsTTP-699 S | UGGAGAUUAGCCUGGGCAA[dT][dT] |
| dsTTP-699 AS | UUGCCCAGGCUAAUCUCCA[dT][dT] |
| dsTTP-830 S | AACUCCUGAUCUCAGGUAA[dT][dT] |
| dsTTP-830 AS | UUACCUGAGAUCAGGAGUU[dT][dT] |
| dsTTP-973 S | GCACUUUGCUCUGCAGAUU[dT][dT] |
| dsTTP-973 AS | AAUCUGCAGAGCAAAGUGC[dT][dT] |
| dsControl S | ACUACUGAGUGACAGUAGA[dT][dT] |
| dsControl AS | UCUACUGUCACUCAGUAGU[dT][dT] |
